# More than participatory? From ‘compensatory’ towards ‘expressive’ remote practices using digital technologies

**DOI:** 10.1177/14687941231165882

**Published:** 2023-03-23

**Authors:** Susanne Börner, Peter Kraftl, Leandro L Giatti

**Affiliations:** School of Geography, Earth and Environmental Sciences, 150748 University of Birmingham, Birmingham, UK; School of Public Health, 28133 University of Sao Paulo, São Paulo, Brazil

**Keywords:** Participatory action research, COVID-19, digital geographies, marginalised groups, WhatsApp, youth, digital methodologies, participant expression, knowledge co-production

## Abstract

Based on the shift from face-to-face participatory action research (PAR) with groups in situations of vulnerability to digital methods during COVID-19, we reflect on how we can go beyond *compensating* for the physical absence of the researcher from the field. We argue that instead of simply aiming to replace face-to-face research with a digital equivalent for maintaining ‘participatory’ and ‘inclusive’ research practices, remote practices have the potential of being *more-than* compensatory. We suggest that when producing multi-method digital approaches, we need to go beyond a concern with participant *access* to remote practices. By rethinking remote PAR in the light of *expressive* rather than *participatory* research practices, we critically reflect on the (sometimes experimental) *process* of trying out different digital research method(s) with Brazilian youth in situations of digital marginalisation, including the initial ‘failures’ and lessons learned in encouraging diverse forms of participant expression, and ownership using WhatsApp.

## Introduction

The COVID-19 pandemic has marked a point of disruption in the status quo of research practices of qualitative researchers worldwide, and especially those engaged in participatory action research (PAR). Before the pandemic, most researchers had relied on face-to-face partnerships with local communities and partner organisations for collaborative data collection and analysis (Valde and Gubrium, 2020; Hall et al., 2021). For those researchers engaged in the co-production of knowledge in conditions of socio-economic and digital vulnerability, the pandemic presented additional challenges in maintaining personal relations and interactions. As (Nguyen et al., 2021: 2) observed, ‘vulnerable groups are likely to be least prepared to manage shifts necessary during the pandemic and digital inequalities are one way that the crisis might disproportionately impact those groups’. The digital exclusion of marginalised groups under COVID-19 has exacerbated emerging ‘epistemic injustice’ (Liegghio and Caragata, 2021:150), also referred to as ‘epistemic violence’ (Liegghio, 2016; in Liegghio and Caragata, 2021: 150), as well as ‘cognitive exclusion’ (Giatti, 2022: 2). These terms can also be understood as ‘the dehumanization of marginalized groups and peoples through the exclusion of their knowledges and ways of knowing’ (Liegghio and Caragata, 2021: 150).

Meanwhile, even during the pandemic, researchers held onto the potential of PAR research to mitigate inequities ‘through transforming the conventional unilateral relationship between science and society into a [fairer] and more symmetrical process’ (Giatti, 2022: 1). Hence, it is not surprising that much of the recent academic literature on PAR research practices during COVID-19 has focused on how researchers have aimed to *compensate* for their physical absence from the field while attempting to maintain ‘participatory’ and ‘inclusive’ research practices (Auerbach et al., 2022; Hall et al., 2021). By contrast, this present paper aims to move beyond those reflections to outline how – especially – digital methods may extend beyond compensation: how they might enable forms of *expression* that may, ultimately, encourage researchers to reconsider what they mean by ‘Participatory Action Research’. The paper also explores in far greater detail the very (messy, complex) process of putting digital research approaches into practice when working with remote, marginalised communities, including the false starts, failures, experiments and attempts to ‘fit’ such approaches with the capabilities, resources and circumstances of diverse individual participants.

Although some researchers were forced to put their research on hold or entirely suspend their projects during the pandemic, there are numerous accounts of how researchers have adapted their research to compensate for their physical absence from the field (Hall et al., 2021; Liegghio and Caragata, 2021; Marzi, 2021; Savadova, 2021; Woodward et al., 2020; Cuevas-Parra, 2020; Birkenstock et al., 2022; Dodds and Hess, 2020; Colom, 2022). For instance, (Savadova, 2021:1) assigned ‘proxy’ researchers in situ, thereby ‘minimising or completely removing the need for the researcher to physically be present in the field’. Moreover, Liegghio and Caragata (2021) describe the rationale for choosing photo-voice over video interviews to maintain relationships at a distance while Marzi (2021) explains how participatory video-making was developed as an alternative to co-produce research without face-to-face contact. The different experiences of PAR researchers during COVID-19 have raised questions of power and ownership – often calling into question the possibility of conducting research entirely online while *simultaneously* shifting ownership to local communities and research partners (Marzi, 2021; Hall et al., 2021).

Remote research with groups in situations of vulnerability has however raised questions related to digital access for marginalised populations and the accessibility of digital methods (Kustatscher et al., 2020; Liegghio and Caragata, 2021; Savadova, 2021; Marzi, 2021; Colom, 2022; Woodward et al., 2020). This has brought to the fore questions of rights and equality in relation to internet access, digital literacy, skills and access to technology (Kustatscher et al., 2020). With an increase in the use of smartphones also by vulnerable populations, such devices have been presented as a solution for connecting with participants remotely ‘to investigate participants’ experiences of and perspectives on their everyday lives’ (Marzi, 2021: 3).

The use of social media by vulnerable groups (and the study thereof) however did not start with the pandemic. Especially for young people in urban peripheries, social media was already part of their reality as a means of communication, friendships, social inclusion, empowerment and a way of modifying traditional social ties (Patulny and Seaman, 2017; Décieux et al., 2019). Moreover, across the different geographical subdisciplines, researchers have increasingly explored ‘digital methods’ (Ash et al., 2018a) and the so-called ‘digital turn’ (Ash et al., 2018b), studying digital tools and interfaces and exploring user experiences, practices and responses (Ash et al., 2018a; Ash et al., 2018b). Pre-COVID-19, researchers also began to recognise the potential of smartphone-based methods to study disadvantaged groups (Sugie, 2018). Indeed, those using qualitative research methodologies have integrated the use of smartphones, including digital and video-diaries (Volpe, 2018; Nash and Moore, 2018) and smartphone apps (Hadfield-Hill and Zara, 2018; Do and Yamagata-Lynch, 2017), while broader studies have interrogated children’s ‘presence’ with/in social media in diverse ways (Kraftl, 2020). Previous research has, for instance, demonstrated ways in which the use of social media can shape alternatives for social participation, such as in urban and spatial planning processes (Lin and Kant, 2021). However, despite their digital nature, most of these pre-pandemic digital methodologies generally relied heavily on face-to-face research, whether for participant recruitment, the building of trust, participant training, data collection and/or collaborative data analysis.

Notwithstanding the above developments in the use of digital technologies, during the COVID-19 crisis, in the face of national lockdowns and a ‘digital divide’ (Warner-Mackintosh, 2020:2 in Hall et al., 2021:10), remote (PAR) research became mostly *compensatory* in nature, aiming to replace face-to-face research with a digital equivalent. Hence, most of the studies about the shift of PAR to the digital have addressed issues such as maintaining social relationships at a distance, access to technology, equal participant collaboration, ownership, researcher control and power relations in the physical absence of the researcher from the field (Hall et al., 2021; Marzi, 2021; Auerbach et al., 2022). This work includes discussions about ‘innovative methods to bridge these [digital] divides and maintain the close social ties which allow for participant collaboration and reflection on research issues’ (Hall et al., 2021: 10). It is, in other words, about attempts to mimic PAR approaches, principles, methods and forms of relationship-building through the digital.

However, to date, there has been surprisingly little discussion about the extent to which remote practices – especially when engaging marginalised groups – have the potential of being *more-than* compensatory in terms of creating ‘digital access’, by enabling new opportunities for participants to be heard and/or seen. Whilst some scholars explore the potential of media such as WhatsApp for ‘inclusive conversations’ (Colom, 2022: 9), the question to ask may not be to what extent remote PAR designs enable good and inclusive participatory practices to compensate for face-to-face interactions, but perhaps still more ambitiously and generatively, to what extent remote research practices enable the *expression* of participants*?* How can we rethink key concerns of PAR – co-production, ownership and power – in the light of *expressive* rather than *participatory* research practices? And ultimately, if we talk about expression rather than participation, does this imply a move away from PAR? And if yes – to what?

In addition, few studies reflect on the actual *process* of trying out different digital research method(s) to find methods that are the ‘best fit’ with participants and their own contexts and capabilities (from access to mobile/smartphone data to their confidence expressing themselves via different modes, such as images or text). This includes exploring initial ‘failures’ and learning from what is and what is not working well to make further adaptations during the research process (Davies et al., 2021). Hitherto, the adaptation of methods during COVID-19 has been generally been presented as a single choice (the move from ‘in-person’ to ‘online’) rather than as an actual process (Liegghio and Caragata, 2021). We argue that in a research environment which values research successes – and ultimately, the data you get – and which likewise condemns failures (Harrowell et al., 2018; Davies et al., 2021), valuing the learning process is fundamental. Although these experiences may divert from the intended and validated path in a research proposal, they might lead to forging new understandings of what really matters and, hence, enable new, unanticipated, potentially more diverse forms of expression by and with research participants.

In this article, we will develop our argument based on (remote) research with approximately 40 marginalised young people in the urban periphery of São Paulo during COVID-19. The research presented in these vignettes is part of the project ‘Building resilience in the face of nexus threats: local knowledge and social practices of Brazilian youth (NEXUS-DRR)’^1^. The research had been designed pre-COVID-19 as a hybrid PAR project with both face-to-face and digital components (described below). After a temporary suspension, the research methods were adapted during COVID-19 and the project was conducted mainly remotely through WhatsApp using multi-modal methods. The remote research activities were developed using an experimental approach including a *trial* phase to learn from early failures and from what worked well to then develop and refine the approach during the *consolidation* phase. In developing remote research practices, our initial intention was – as with much previous work – to be participatory and inclusive to compensate for the lack of face-to-face interaction. However, lessons learned over the research process as well as concerns about producing ‘useful data’ made us rethink our understanding of ‘what matters’ (Horton and Kraftl, 2006) in the research process. We gradually opened to diverse approaches to give participants more ownership in their forms of *expression*, although some of the chosen channels of communication (e.g. text message over video) were not always participatory or inclusive in the traditional sense of PAR. However, letting participants decide on their preferred way of communication meant giving ownership and power back to the participants, thereby legitimising the participatory approach (Wallerstein et al., 2018).

In the following, we briefly give an overview of the project and the process of developing (remote) PAR research during COVID-19 before moving on to a discussion a reflection on suspending versus adapting the project during the early days of COVID-19. We then discuss the process of adaption, including initial ‘failures’ and lessons learned, which led to a multi-method digital approach to encourage different forms of participant expression.

## The project: NEXUS-DRR

The project NEXUS-DRR had the objective of exploring young people’s local knowledges and social practices related to food-water-energy scarcity in the municipality of Franco da Rocha, located in the São Paulo Metropolitan Region, Brazil. It sought to situate these knowledges and practices in the context of climate risks such as flooding and landslides, and to identify pathways for integrating youth knowledge into public policies and education for resilience. The principal investigator (PI) relocated from the UK to São Paulo in October 2019 for the 2-year outgoing phase of her Fellowship.

The first scoping field visits in the community took place between October and December 2019. During this time, a partnership was established with local stakeholders such as the Civil Defence and the Secretariat of Social Assistance in Franco da Rocha, both of which were able to facilitate collaborations with local youth groups. In agreement with the local stakeholders, the project was conceived in the format of a university extension course offered through the School of Public Health at the University of São Paulo. The course aimed to engage young people aged 12 to 18 in reflection on issues related to access to food, water, energy, urban development, climate change and disaster prevention. Formal enrolment in the extension course was however not a prerequisite for participating in the research. Rather, accrediting the research activities through an extension course format was thought to provide benefits to the participants by receiving a certificate from the University of Sao Paulo. Enrolment in the extension course furthermore did not coerce young people to make their data available as part of this research as they could withdraw their participation from the research project at any time with no impact on their enrolment in the course.

Between December 2019 and February 2020, participants were recruited through youth groups in two Social Assistance Reference Centres (CRAS), a public service benefitting families in situations of socio-economic vulnerability who were dependent on food aid and social support. The two CRAS were selected based on their location in disaster risk areas. The first CRAS, Vila Bazú, was located closer to the city centre of Franco da Rocha in the proximity of the river and in an area prone to flooding. The second CRAS, Lago Azul, was based in a remote neighbourhood in the hills with a high risk of both seasonal flooding and landslides.

Participants were recruited with support from the CRAS social assistants through the database of registered families in conditions of vulnerability. This included inviting young people who already attended CRAS activities, such as weekly youth clubs, as well as an ‘active recruitment’ of new young people through visits to families’ homes and local schools. In addition, information days were held for young people and their parents to learn more about the project and to help young people and their parents fill the informed consent forms for participation in the research.

The project had received ethics clearance from the ethics committees of from the ethics committees of the University of Birmingham, the University of Sao Paulo as well as the EU including an online research component using smartphones which had been envisaged pre-pandemic at the beginning of the project. To ensure safeguarding, all data collected from young people was made anonymous for reporting and publication purposes. All data was moreover treated confidentially except where potential child safeguarding issue was identified. Participants were also strongly advised to avoid any situations of risk, especially when conducting smartphone activities (e.g. when taking photographs) and they were advised to immediately report risk situations.

The project had been designed pre-pandemic based on the educational philosophy of Paulo Freire (2000), seeking to involve young people in collaborative research and reflective learning (Giatti, 2019; Börner et al., 2020) using a participatory approach. The Freirean praxis is based on ‘dialogical’ activities and a reciprocal process of collective learning (Wallerstein et al., 2017), which dissolves the boundaries between teachers and students (or researchers and participants), as they also learn from their students (participants) through a process of knowledge co-construction. By researching *with* youth and engaging them as co-producers of knowledge through a process of (1) problem posing, (2) critical dialogue, (3) solution posing, (4) developing action plans, we hoped to develop a better understanding of young people’s views, everyday realities and needs (Bradbury-Jones et al., 2018) and to achieve epistemic justice through the ‘intentional use of methods and methodologies that are inclusive of marginalized knowledges and ways of knowing’ (Liegghio and Caragata, 2021:150). This approach required a close interaction with the community including participatory activities such as youth-led neighbourhood walks, participatory GIS mapping (Carvalho et al., 2021), focus groups and photo-voice activities (Foster-Fisherman et al., 2010). Data analysis was conducted using Qualitative Content Analysis, coding data according into different themes using the software NVIVO.

The research project was integrated into the educational programme of CRAS Vila Bazú and CRAS Lago Azul on a weekly basis. Face-to-face research started in February 2020 in the two CRAS, including initial trust-building workshops, a participatory mapping workshop and a youth-led community walk and reflective activity. With the first lockdown in São Paulo in mid-March 2020, research activities had to be suspended in the early stages. Field research only resumed online in November 2020 and the University of Sao Paulo allowed accrediting the research project as a now remote university extension course. Given its widespread use amongst Brazilian youth, we used WhatsApp as the main means of communication. The online research was conducted between November 2020 and June 2021 through three WhatsApp groups. When using WhatsApp as a research tool, participants were explicitly advised (through adapted informed consent forms and at the beginning of the WhatsApp interactions) that the WhatsApp conversations were part of the research process. We also offered participants to engage in 1-1 WhatsApp chats with the researcher if they felt more comfortable with individual interactions instead of posting in the group chat. As participant participation fluctuated over the research period and some of the young people only participated in the face-to-face workshops but not in the remote research component, the number of participants was not constant. Overall, 33 young people participated in the WhatsApp groups on a regular basis in addition to roughly 10 additional participants that joined several of the face-to-face activities. Ultimately, 15 of the participants successfully completed the remote extension course with an attendance rate above 75% and received an official certificate from the University.

Research activities included weekly synchronous and (a) synchronous activities on WhatsApp such as discussion groups, photo-voice activities, participatory video as well as practical activities. After a temporary return to the UK during the peak of the COVID-19 crisis in Brazil, the principal investigator resumed in person field research in São Paulo between October 2021 and January 2022 to hold face-to-face follow-up meetings with key stakeholders and some of the participants.

The focus of this paper will be on the digital research component to examine in a more sustained way the practical and ethical implications of moving beyond compensation to expression in (online^2^) research with vulnerable groups.

## The early days of the COVID-19 pandemic: project suspension versus adaptation

Caught by the COVID-19 pandemic in the early stages of field research, the first national lockdown in Brazil in March 2020 brought considerable uncertainty regarding the future of the research project. We felt conflicted regarding our ethical responsibility as researchers conducting research with vulnerable communities in times of uncertainty, crisis and stress (Liegghio and Caragata, 2021; Hall et al., 2021). For instance, we questioned how we could continue conducting research on issues such as food insecurity in times of crisis where many families had lost their jobs and suffered from a heightened stress on their livelihoods – even if our research was aiming to address some of these challenges. Although we did not want to abandon pre-existing ties with the community and cancel planned research (Hall et al., 2021), we felt that that researcher-participant relations were not yet sufficiently strong to shift to one-to-one remote interactions in such a time of stress.

To expand on the above: before the first lockdown, the PI had only conducted three participatory workshops with approximately seven participants at CRAS Vila Bazú and one participatory mapping session with a group of approximately 20 participants at CRAS Lago Azul. Although the PI had also participated in trust-building activities such as attending a youth party at the social centre, there was little continuity in terms of the participants that attended these events and the workshop sessions. Even if the institutional structure of the youth groups at CRAS had been pre-existing, most of the participants in our project had been recently recruited for the research and most of the young participants did not have any long-standing relationship with the social assistants at the social centres. In March 2020, we made a first ad-hoc attempt at moving research activities with the participants from CRAS Lago Azul online through a WhatsApp group chat and individual interactions. However, local partners were overwhelmed by the COVID-19 crisis and there was a lack of coordination with CRAS regarding how activities would be managed. The response rate from the group chat was very low and the PI was only able to engage in sporadic individual interactions with some of the young people during the first month of lockdown (Börner et al., 2020). Moreover, interactions felt extractive and concerns about conducting research with vulnerable youth in a time of crisis as well as seemed to outweigh the perceived benefits.

Another key challenge at the beginning of the COVID-19 pandemic was the lack of access to digital technology – both among the CRAS staff and the young participants – including access to smartphones and the internet. Hence, setting up a structured remote research process rather than ad-hoc interactions seemed impossible. CRAS staff initially lacked access to work smartphones and laptops while struggling with understaffing, since most of the staff were dealing with community request related to emergency food aid and social assistance. In addition, some of the young people that had participated face-to-face had no or very limited access to smartphones, as in the case of those using a family phone. Only few of the older participants had their own devices but often no regular access to the internet. A few months into the pandemic, this digital vulnerability was alleviated to an extent by government programmes providing young people with prepaid data packages to be able to follow classes remotely. However, most participants only had access to old smartphones and not all young people were able to download apps requiring a larger memory capacity such as Zoom. All of these considerations meant that we had to engage in ongoing reflections about whether to continue suspending the project or (how) to adapt it.

During the suspension phase of the project, the PI continued sporadic interactions with key stakeholders such as the Civil Defence and the CRAS staff to monitor the COVID-19 situation and a possible restart of fieldwork. Maintaining these personal relations through WhatsApp was an essential prerequisite for restarting fieldwork online in November 2020. By then, both CRAS had adjusted to the new digital reality and were planning to host their youth groups remotely. Hence, they suggested restarting research activities by using WhatsApp groups that would be set up and co-managed by the CRAS.

WhatsApp is available for Android, iOS or KaiOS smartphones with more than one billion users across 180 countries (Colom, 2022). In Brazil, WhatsApp is the most widely used communication technology and accessible at a relatively low cost and is seen as a ‘safe’ mode of communication given end-to-end encryption when messaging. Despite the challenges of digital vulnerability described above, the existent embedding of WhatsApp in participants’ everyday lives (Colom, 2022; Woodward et al., 2020; Mavhandu-Mudzusi et al., 2022) enhanced its potential of being a tool for overcoming epistemic injustice by enabling a dialogue about concerns and knowledge among vulnerable youth and academic researchers (Santos et al., 2016; Hall and Tandon, 2017).

## Adapting the project: learning from failures and the importance of being experimental

WhatsApp was a key communication tool both for participant recruitment and communication during the research process. The two CRAS oversaw participant recruitment using WhatsApp messages and phone calls to contact families registered with the both social centres. It is important to flag that not all the participants involved in the digital component had participated in the pre-pandemic face-to-face workshops and, in addition, CRAS recruited new participants.

Following participant recruitment, we engaged in an *experimental process* using WhatsApp to develop a remote multi-modal research approach which would be the ‘best fit’ for achieving epistemic justice (Liegghio and Caragata, 2021:151), aiming at inclusive and participatory PAR. It is important to emphasize that – as well as being experimental – we based our methodological adaptations on dialogues with our collaborative networks, a review of emerging literature on remote research methods in the context of the pandemic, and previous experiences in research using digital technologies for social engagement in predominantly ‘face-to-face’ research. All these contributed to redefining processes and interactions in participatory research (Brydon-Miller et al., 2003; Giatti, 2019).

Without previous experience in using WhatsApp for digital youth research, we found ourselves exploring new terrain. Although we had an initial idea of the issues that we wanted to explore with the participants, we were hesitant about how to go about this remotely while maintaining participant relations online. To deal with anxiety about the unpredictability of the process and participant engagement (Bashir, 2020), we decided that our best bet was to go into the research process with openness and curiosity to design the activities based on what we (and the participants) felt worked best. This openness was also needed for navigating not only our research interests but also the expectations of local stakeholders. Once we had decided on a tentative online format, one of the collaborating social centres was keen on resuming activities as soon as possible. Recognising failure as an inherent part of academic knowledge production (Davies et al., 2021: 1) was essential to see the initial ‘trial’ phase as a learning process and a catalyst for adaptations during the research process.

**Figure 1.**
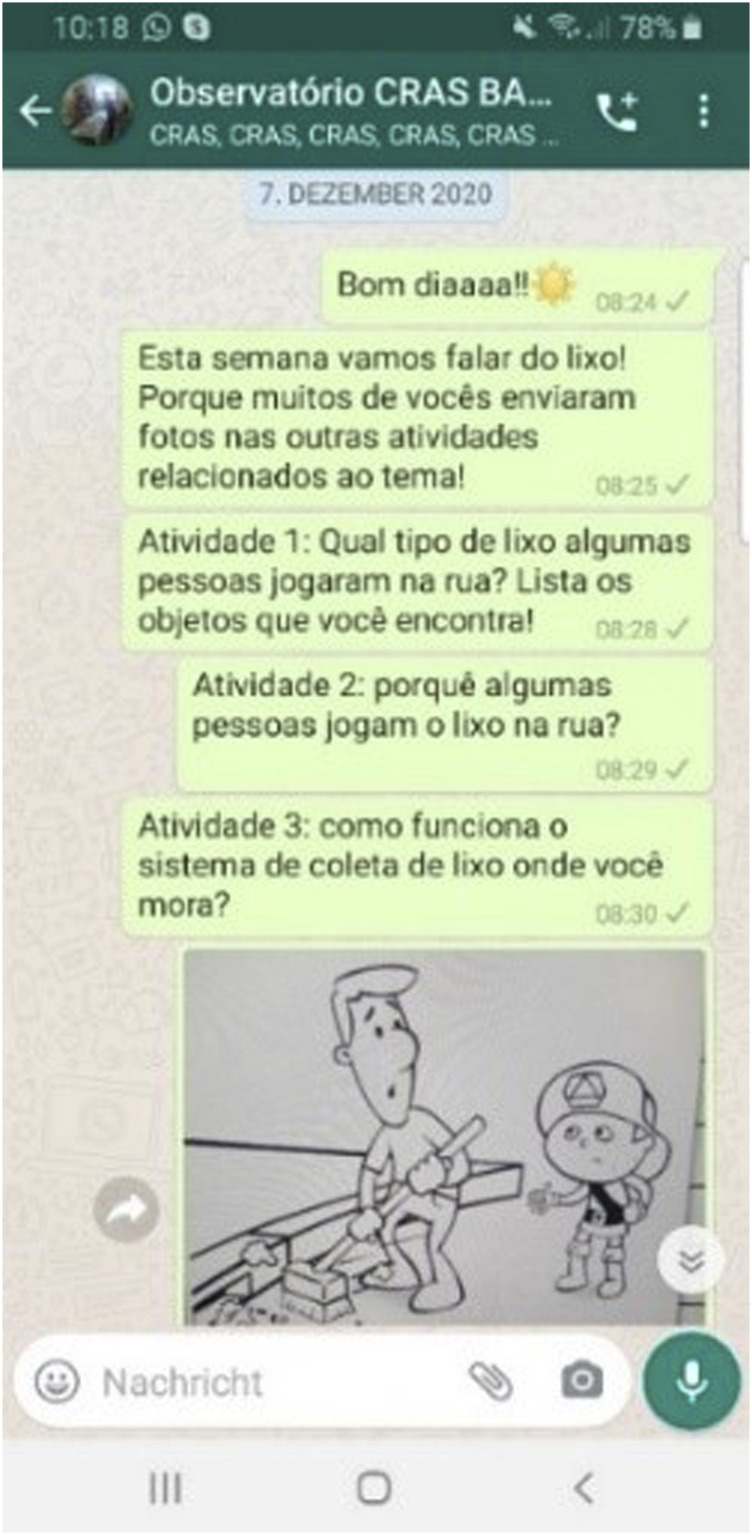
Screenshot of weekly activity including cue for discussion.

To keep participants engaged over several weeks, the PI came up with an initial plan for weekly assignments, although in practice these were adjusted on a week-by-week basis depending on participant participation and interests. These research activities were designed in a way to be engaging, participatory and playful by including different techniques such as written assignments, photo-voice, practical assignments and participant videos that explored young people’s experiences with the access to and use of food, water and energy in a context of disaster risk. Figure 1 illustrates an example of one of the weekly (asynchronous) activities on pollution where we asked participants to report back on different activities during the week to comment on a cue for discussion. The English translation (by the authors) of the activity in Figure 1 is as follows: *‘Good morning! This week we are going to talk about rubbish! Because many of you have sent in photos in the other activities related to this topic. Activity 1: What kind of rubbish did people throw in the street? List the objects you find. Activity 2: why do people throw rubbish in the street? Activity 3: How does the rubbish collection system work where you live?’*

The below reply is an example of participant responses over the course of the week:

*Name: anonymous*

*Age: 13 years old*

*Activity 1: The types of rubbish are: plastic bags, snack wrappings and other types of rubbish.*

*Activity 2: Some people throw rubbish in the street because they have no idea of the consequences that this can cause.*

*Activity 3: The rubbish collection system where I live works like this: sometimes some residents get together to collect the rubbish.*

### Initial challenges: ‘Extractivism’, lack of trust and moral responsibility

On top of the already ‘messy’ nature of PAR research (Marzi, 2021), being thrown into this ‘experiment’ of remote research created additional challenges. One key challenge was research ethics. In charge of supporting us with obtaining written consent from the participants and their care givers, the CRAS struggled to collect all consent forms before the start of the remote activities (although we eventually managed to get the proper consent in all cases). Another challenge was that the CRAS kept adding new participants to the WhatsApp group. Ultimately – over the entire project – 30 youths participated through three parallel WhatsApp groups with varying degrees of frequency. Although a more committed core group emerged over the course of the activities, not clearly knowing ‘who was who’ made it difficult to build trust remotely, especially with the less engaged participants. Only some of the virtual participants had joined the face-to-face workshops at the start of the project before the pandemic. Some of the less frequent virtual participants remained only a phone number which we could not fully connect to a face. Moreover, newly added participants initially lacked clear information about the project (and had to be informed individually) and had also missed out on previous discussions. Since most of the young people used their parents’ phones – some of whom kept changing their numbers – it was initially difficult to identify participants until we asked them to include their name and age when replying to messages in the chat (as illustrated by the above responses to the weekly activities in Figure 1). Starting their interactions by saying, ‘my name is Marcio and 14 years old’ made communication appear rather instrumental and, especially, even more ‘extractivist’ in the sense of creating a top-down teacher-student dynamic between the researcher and the participants rather than horizontal relations. The nature of these asynchronous messages also inevitably reduced spontaneous and dynamic interactions between participants as young people responded to the weekly activities in ways likely akin with how they responded to a school assignment (i.e. seeing the PI as an ‘authority’ adult or even ‘teacher’ during the weekly research/university extension activities). This reduced the space for ‘group reflexion and deliberation’ (Colom, 2022: 10) and made the process of ‘bonding as an already organised group’ (ibid: 10) challenging. We were also concerned that this focus on ‘information extraction’ (Liegghio and Caragata, 2021: 151) might lead to bypassing the ‘relational element’ (ibid:151) of PAR, since there was little room for connecting with the participants on side-issues such as their everyday concerns.

In terms of achieving high participant response rates, it soon became clear that asynchronous weekly activities worked better than synchronous activities where participants had to connect at a certain time every week. The inability of most of the participants to join meetings at a set time, limited access to phones and shyness to discuss issues in a face-to-face group setting online were only some of the limiting factors. One of the participants explained her experience: *‘In the beginning it was a little bit difficult to participate in the conversations, I didn’t have a mobile phone at the beginning and sometimes the time didn’t match with my schedule, but then it got easier’.* Some of the participants also chose to send some of their answers individually to the researcher rather than engaging in the group chat if they felt uncomfortable sharing experiences in the group (Barbosa and Milan, 2019).

Moreover, we faced the challenge that some of the mothers seemed more interested in participating than their children. We had to find more inclusive ways of encouraging family participation, for instance, by asking parents to make short videos together with their children. However, we had to make it repeatedly clear to parents that the WhatsApp group aimed to engage their *children* rather than inviting adult participation. As we began to consider the above, we also had to deal with adverse reactions from some of the parents. In particular, one of the mothers questioned the purpose of conducting research activities on environmental issues when some of the families were suffering from severe food insecurity and had other priorities to deal with. In addition, the above described challenges to connecting to some of the participant virtually created barriers to discussing sensitive topics (Santana et al., 2021). It is also important to acknowledge that even if the wider families were not overtly involved in the research process, family (power) relations and practices may still have an influence on young people’s engagement (Manney, 2013).

Again, these reactions made us question our ethical responsibilities around conducting research on issues such as food insecurity during times of crisis. We also started reflecting on ways of addressing sensitive topics without creating distress and making research more fun – and ultimately expressive – by looking at positive aspects of the environment and not only at the problems. This also speaks to reflections on whether (and how) research can be adapted to include (creative) methods which encourage reflection while taking care of psychological participant wellbeing (Lazarte et al., 2020; Pacheco and Zaimagaoglu, 2020; Hall et al., 2021). We examine these considerations in more detail in the next two sub-sections of the article.

### Learning from the ‘failures’ of the trial phase: curiosity, mutual trust and humour

The above challenges show that developing remote methods was not only a one-off choice – as it has often been presented in previous publications – but an experimental *process* which required a lot of curiosity and willingness to learn from and with community stakeholders and the participants. The initial challenges and ‘failures’ in the process of engaging remotely with the young people were fundamental for helping us refine our approach to develop a multi-modal method based on participants’ different needs and the ‘best fit’ with their lives. After the first ‘trial’ phase, we continued research activities in two new WhatsApp groups in a more structured manner.

One of these new WhatsApp groups was hosted virtually by CRAS Vila Bazú (CVB) and the other one by CRAS Lago Azul (CLA). At CLA, a 22-year-old social assistant was committed to leading the weekly group activities online during the regular meeting time of the CRAS youth groups on Wednesday afternoon (approximately 1 h). The engagement of the social worker was fundamental since she actively supported the group activities by sending out reminders and actively participating in the activities. At CLA, we opted for a synchronous format of online group discussions on WhatsApp with the possibility of asynchronous participation in a weekly activity for those who were not able to join ‘live’. At CVB, we opted for asynchronous weekly activities which were sent every Monday morning with the opportunity for interaction during the week. These included input for discussion such as short thematic videos, pictures, audio recordings or questions for discussion. As a result, thematic activities at both CRAS were developed similarly but with some differences, since synchronous versus asynchronous activities also led to a different dynamic between researchers, CRAS staff and young people (summarised in Table 1).

**Table 1.** Overview of thematic activities in the trial and consolidation phase.

	Week 1	Week 2	Week 3	Week 4	Week 5	Week 6	Week 7	Week 8	Week 9	Week 10	Week 11	Week 12	Week 1 3	Week 14	Week 15	Week 16	Week 17	Week 18
Asynchronous activities CRAS Vila Bazú Group 1	Brainstorm environment	Introduction to disaster risk	Water usage	Participatory activity about ‘everyday life’ using video	Waste and pollution	Environmental risks	Climate change	Participatory agenda-setting for next topics	Online community journal: Creative exercise (text and photo)	Recycling and reusing	Practical activity: reusing	Rain and flooding	Ideas for change: neighbourhood in 5 years	Video calls with individual participants	Food diaries	Community-based food production	Community gardens	Healthy food
Asynchronous activities CRAS Vila Bazú Group 2	Virtual getting to know each other	Brainstorming on ‘environment’	History of flooding in Franco da Rocha and places I like and don’t like where I live	Access to (healthy) food and local food production	Water usage and discussion of favourite places	Brainstorming: a city for young people?	Participatory risk mapping (invited researcher)	Youth participation and youth voices for change (invited youth activist)	Earth day and environmental solutions	Environmental solutions part II	Mobility, leisure and we--being	Mobility, leisure and wellbeing part II	Transport and mobility	Individual projects of choice (e.g. air pollution, transport, flooding)	—	—	—	—
Synchronous activities CRAS Lago Azul (with asynchronous component)	Virtual getting to know each other	Brainstorming on ‘environment’	Introduction to disasters with a focus on landslides	History of flooding in Franco da Rocha	Introduction to climate change and virtual water	Healthy lifestyle, wellbeing, access to water and healthy food, greening of cities	Earth day: why are nature and the planet important for you?	Participatory risk mapping (invited researcher input)	Youth participation and youth voices for change (invited youth activist)	Impact of covid-19 on education and mobility	---------(Holiday)	CRAS activity	Access to healthy food	Use of backyard and gardens	CRAS activity	Transport and mobility	—	—

Overall, one important lesson learned from the trial phase was the importance of establishing positive emotional connections to create more horizontal researcher-participant relationships for collaborative learning whilst doing research *together* (Hadfield-Hill and Horton, 2014). To this purpose, it is fundamental to reiterate the importance of legitimacy and (mutual) trust building (Christopher et al., 2008; Wallerstein et al., 2018) to support remote relationship-building. We therefore asked each of the participants to send a short video or photo with their name, age and a short description such as hobbies and interests while also sharing a short video and photo-activity about ourselves. In addition, we offered an introductory video call. However, only few participants were able or willing to participate due to patchy internet, insufficient data for online communication or conflicting engagements such as school work.

**Figure 2.**
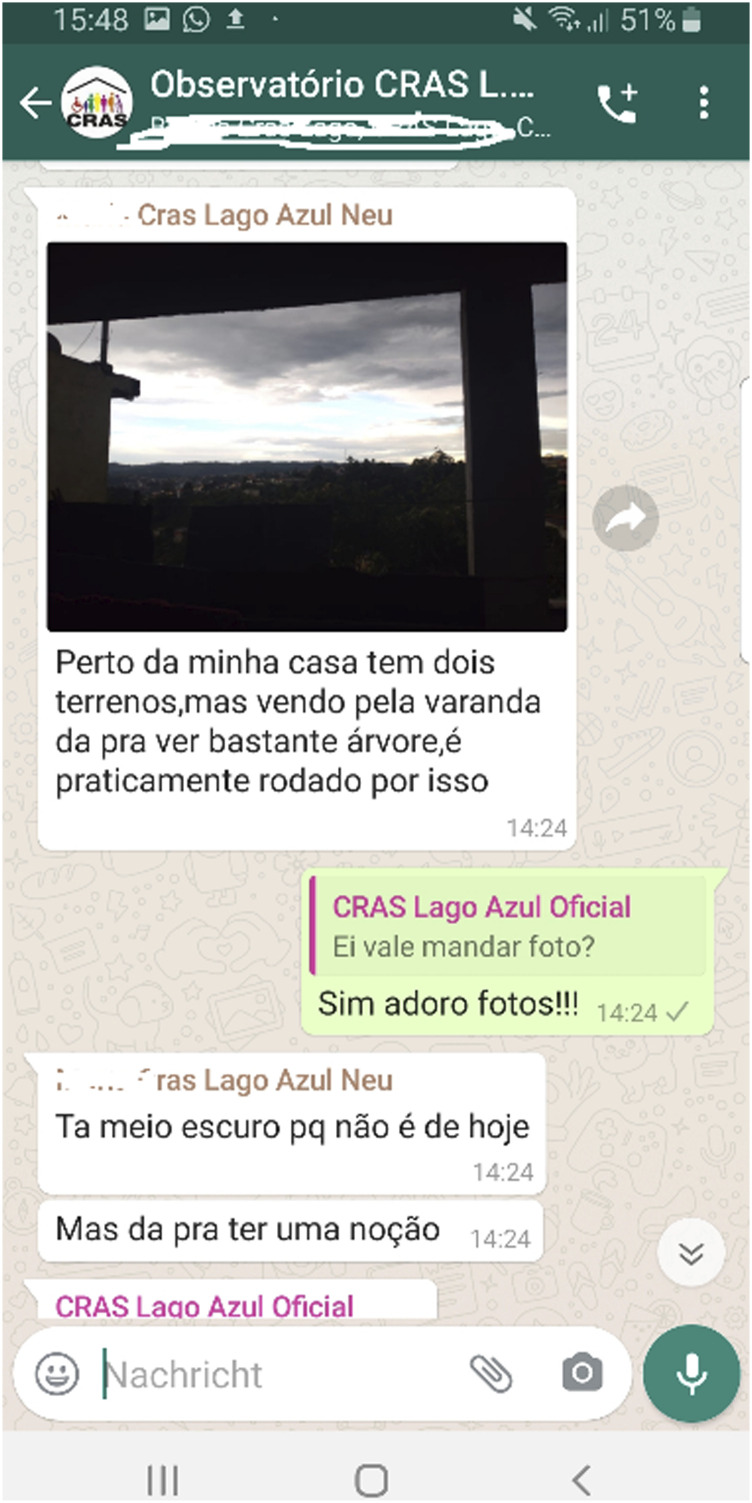
Example of group reflection during synchronous interaction. Part of the translated discussion is shown in the WhatsApp screenshot together with the picture that was commented on in the discussion.

We also aimed to create both asynchronous and synchronous spaces for more ‘group reflexion and deliberation’ (Colom, 2022:10) by encouraging youth to respond to each other’s comments, ask questions and interact – with a mixed success. When asked how we could improve interaction, one participant suggested ‘*I recommend mentioning the participants when you have any questions [@name], try to talk and understand each other’s side. That helps a lot!*’*.* At the same time, young people participating in the asynchronous group chat perceived the overall lack of interaction *among* the participants as a major challenge; as one of them joked: ‘*It seemed that there was only me*!’. Overall, opportunities for bonding, deliberating and interacting were more available in the *synchronous* group activities, as illustrated by the below example of a synchronous group reflection on topics related to environmental issues Figure 2.

*PI: Why did you think of “mato”?**3*

*Participant: Because it is usually associated with plants or nature and that’s what we have here.*

*PI: Very cool, so I think it’s very important to include it!*

*PI: I grew up next to a forest but maybe it's different from the bush here.*

*CRAS social assistant: Can I send you a photo?*

*Participant: <picture > Near my house there are two plots of land, but from the balcony you can see a lot of trees. And it’s practically surrounded by it.*

*PI [commenting on message from the CRAS social assistant]: Yes I love photos!!!*

*Participant [commenting on picture that she sent]: It’s a bit dark because I didn’t take it today. But you can see what it's like.*

*CRAS social assistant: What a great view. There are many trees!*

*Participant. The sky looks beautiful.*

*CRAS social assistant: Yes!*

Creating and deepening trust meant blurring researcher-participant boundaries by giving back, not only in terms of technical content but also in researcher reciprocity by sharing everyday experiences and realities (Swartz, 2011). Again, this process led to some practical, emotional and ethical considerations. By mutually sharing stories and photos about everyday routines and experiences (e.g. access to food, urban mobility), these kinds of interactions sparked participants’ curiosity. During synchronous interactions, young people were keen on learning more about food or urban mobility in Germany (the country of origin of the PI) or the UK. Participants felt encouraged to actively ask questions while helping them reflect on their own everyday environments in a different way. Moreover, as a foreign researcher in Brazil, the principal researcher was able to ask ‘obvious’ questions to make young people explain and reflect upon their everyday realities in a way that was defamiliarising but often generative.

At the end of the project, some of the participants described the experience in the following way: ‘*The one word that describes [the project] is ‘exciting’. It was a very different experience, out of the ordinary’.*

**Figure 3.**
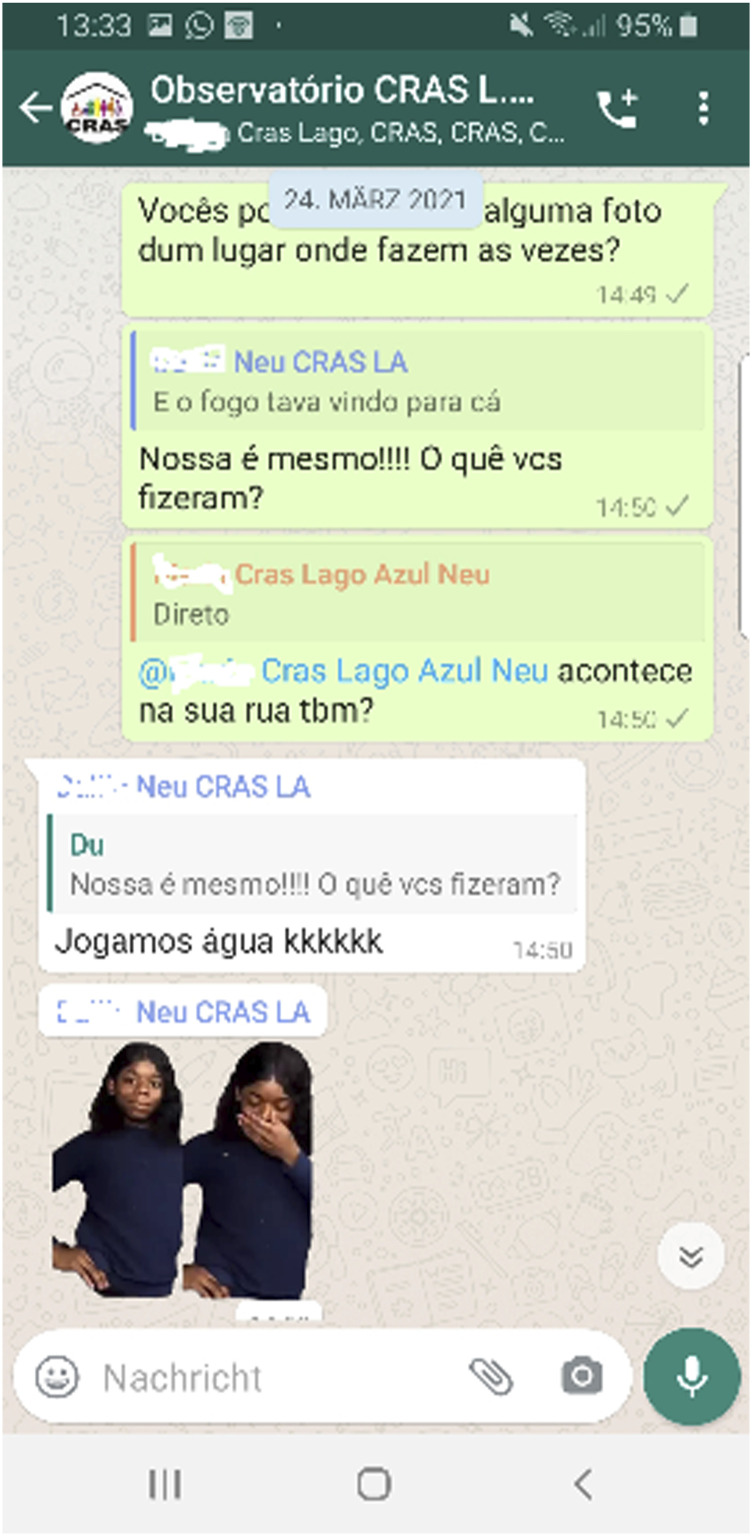
Using humour to address sensitive issues, such as the risk of wildfires.

In the process, we discovered humour as an important means of talking more ‘lightly’ about problematic issues such as urban pollution or food insecurity. This included using funny GIFs to end a conversation on wildfires in the neighbourhood conversation on a light-hearted note (see Figure 3 Translation by the authors: *Interviewer: What did you do* [*about the fire*]*? Participant: We threw water. (GIF),* thereby making conversations around sensitive issues more comfortable (Dodds and Hess, 2020). As illustrated by the below conversation in Figure 3, we also tried looking at the positive aspects of participants’ local environments – despite complex problems. By adopting a perspective *beyond* a mere focus on problems and guiding conversations from ‘despair to joy’, we sought to navigate the stigmatisation of peripheral urbanisation processes in the global South (Allen, 2022).

### Beyond compensation: encouraging expression through multi-modal methods

Trust-building and more horizontal researcher-participant relations were a key element for participant engagement. However, despite improved trust, the challenge of being more dialogic, fun and engaging remained. We therefore kept experimenting with content and techniques – *initially* with the objective of creating methods in the spirit of PAR to ‘compensate’ for our physical absence from the field, but increasingly with the objective of facilitating participants to *express* themselves in ways that felt comfortable and meaningful for them. Going beyond issues of accessibility, this meant exploring the ways in which participants felt at ease expressing themselves in *diverse* modalities – whether in a one-to-one approach using text or audio, in written group discussions, or through pictures and video. Exploring different modes of expression also meant providing participants with *options* rather than insisting on ‘a method’, thereby giving participants ownership in deciding on their preferred channel of communication for expressing their ideas and best fitting their familial/technological circumstances. This aligns with reflections by Clark and Moss (2011) on developing new practical and imaginative ways of listening to children’s perspectives through multi-methodological techniques, or what they call the Mosaic approach, to recognise ‘symbolic language’ and enable a choice of ways in which participants can engage. The importance of giving participants ownership over choosing their preferred method of communication is illustrated by the following WhatsApp interaction:

*PI: Would you guys like to do a little video call today?*

*PI: Or we can talk here as well, so that everyone can read it later!*

*Female participant: I vote for the chat hahaha*

Initially, we had prioritised creating ‘interactive’ activities such as making a video diary or practical activities such as making objects from recycled plastic bottles as digital PAR. Overall low response rates for these kinds of ‘creative’ activities and (perhaps surprisingly) a high participant response rate for written activities showed that a focus on *expression* was sometimes preferable to the kinds of PAR ‘activities’ often favoured by participatory, creative and arts-based researchers. This is not to say that there is anything wrong with those latter methods, but that some young people may favour ‘basic’ writing over more ‘elaborate’ methods – whether for technological reasons (speed, data availability) or because they feel more comfortable expressing themselves in this way. As one of the participants explained, ‘*the advantage was that chatting by message is easier, because I’m a bit shy, and even being able to formulate a sentence talking face to face is a little difficult hahaha’.*

Table 2 further summarises participants’ preferences as well as the advantages and challenges for the different engagement methods. It shows the complexity of deciding on a technique, since each approach has certain disadvantages and advantages for creating connection, participant ownership and inclusivity. We therefore reiterate the importance of the *process* of (simultaneously) exploring various methods for participant engagement. Moreover, there is no such thing as ‘the participant’ since the young people involved in the project had very different personalities, digital skills and preferences as well as varying access to mobile phones and internet. Hence, experimenting with multiple methods created diverse spaces for expression, deliberation and reflection. Sometimes, this even led to unexpected forms of participant ‘creativity’, for instance, where participants had the idea to share and comment on Google Streetview images to illustrate their contributions to avoid leaving the house during the COVID-19 pandemic.

**Table 2.** Multi-modal methods and their potential for expression.

Technique	Participant preferences and potential for expression
Text-based activities	Participant preference for text-based interaction or mix of text and audio responses. This includes GIFs and emojis to express sense of humour (‘hahaha’) to react. Ranging from one-word responses (yes/no) to longer text replies to questions. Enabling for shy participants.
Photo-voice	Used by some of the participant, mostly taking pictures taken from the window or in the backyard. Participants were not always able to leave their house to take photos because of COVID-19 restrictions or safety issues and used Google Streetview screenshots instead. Required access to a phone with camera and sufficient internet access to send the pictures.
1-1 interactions using audio/text/video	Conversations often flowed better in 1-1 interactions rather than in the group chat (especially in asynchronous interactions). Some participants only accompanied the chat but did not participate actively. In the group chat, addressing participants individually by their names (e.g. @Ana) stimulated responses in the group. Participants were not always comfortable sharing in the group and sometimes preferred 1-1 interactions.
Group discussions (e.g. using GoogleMeets)	Limited participation in group or 1-1 video calls. Overall preference for text-based interactions. Challenges related to access and connectivity, stability of the internet connection. Little expression of shy participants.
‘Practical’ assignments and ‘digital arts’	Little uptake from participants for creative methods (e.g. making toys from recycled water bottles). Lack of time, material and support for the activity. Overall preference for ‘simpler’ (text-based) methods.
Participant videos	Limited response rate. In terms of trust-building, preference for sending photos over presentation video. Few participants participated in video diary activity to document everyday places or ways to protect the environment, although two sisters produced a self-edited video.
Multi-stakeholder engagement	Engagement of youth activists and researchers to deliver a weekly activity (e.g., input via audio and photos) to encourage participant reflection and spark interest in new topics.
Group discussions (text-based)	Different dynamics depending on **synchronous** versus **asynchronous** discussion **Synchronous:** Real-time exchange and questions, opportunity to make jokes, ability to clarify responses, cues from the social assistant to stimulate the discussion. Prompted more reflexivity and knowledge exchange (e.g., discussion of use of backyard space in Brazil vs the UK). Challenges: Limited access to mobile phones during the set times. Time constraints (interactions limited to 1 hour). Conflicting engagements such as school work, training activities or household chores. **Asynchronous:** Lack of interaction between participants and tendency toward knowledge extraction. Lack of interaction between participants. ‘Classroom style’ responses to questions or an assignment. Advantage: Levelling out of competing demands such as school work or household chores and flexibility in access to phones. Sometimes mixed with synchronous interactions when we caught participants in a ‘chatty moment’ and were able to engage in real-time conversations.

## Discussion and conclusion

This article has argued for a way of considering a move to digital research methods – from or alongside more ‘conventional’ forms of PAR – as not merely *compensatory* but as enablers of multiple forms of *expression*. We have emphasised that attentiveness to the diverse modes in which ‘participatory’ (or otherwise) forms of data are articulated is key, and especially for vulnerable groups (like marginalised young people), whose familial and technological circumstances, as well as whose preferred modes of expression, might be diverse, complex and dynamic. The article has also, therefore, called for and exemplified a greater acknowledgment of some of the practical and ethical concerns involved in the very *doing* of digital research at a distance.

The purpose of this article has been to open out reflections and questions about the use of digital methods that may be useful to other researchers. Alongside scholarship elsewhere around academic failure (Harrowell et al., 2018; Davies et al., 2021) and ‘mess’ as method (Law, 2004), we wonder whether – especially when approaching such methods from a ‘compensatory’ stance – we ironically expect *too much* of those methods, and our participants/co-researchers/any proxies who might replace us in the field (Savadova, 2021). In other words, if there is a sense that digital technologies may ‘replace’ established PAR methods, we – those of us involved in the co-production of research – may feel under too much pressure to be ‘creative’, to be ‘engaged’, in ways that might meet the ‘gold standards’ of PAR. We have tried to argue in this article that a focus on diverse modalities of expression might enable involvement in research that is contextually aware and a better ‘fit’ for participation. Some of these forms of expression may appear conventional, staid even – particularly some young people’s preference for straightforward 1:1 WhatsApp text messaging – but may be generative in enabling effective research that more than compensates for, and may offer opportunities beyond, a lack of face-to-face interaction.

These reflections have led us to consider several questions over the course of the project and our changing engagement with young people. For instance, how can we rethink key PAR elements such as co-production, ownership and power in the light of *expressive* rather than *participatory* research practices? And if we talk about expression rather than participation, does this imply a move away from PAR? And if yes, to what?

Firstly, as we have argued, a focus on expression takes us beyond a concern with ‘access’ (Colom, 2022) – whether to (WhatsApp) technology or to the kinds of capabilities required to participate meaningfully in digital research. Moreover, such a focus takes us beyond the (apparently) simple decision as to whether to move ‘to’ digital methods ‘from’ face-to-face interactions. Instead, we have sought to open out this move as a complex *process*; crucially, doing so (re-)casts research participants as individuals, who have personal preferences around how they express themselves (which, of course, may intersect with ethical concerns such as their ability to access a mobile phone app or other technology). In contrast to those studies that have examined WhatsApp for its communicative value in a slightly extractivist sense (e.g. Mare, 2017; Gibson, 2020), we were particularly interested in how digital forms of engagement that require apparently minimal time, energy or need to reveal much of ‘oneself’ were potentially favoured by participants who wanted to be less visible, or who were quieter, shier or less confident. Indeed, we wonder whether some young people took part in the *digital* research but would not have had the confidence to take part in the participatory research had it been in-person.^4^ Some of our participants furthermore preferred 1-1 interactions with the researcher over engaging in a group setting. This also raises discussions on the ethical principles of WhatsApp group chats (Barbosa and Milan, 2019). Although beyond the scope of this paper, we hence consider that more research is needed to explore the ethical challenges of WhatsApp as a data collection tool (Mavhandu-Mudusi 2022; Barbosa and Milan, 2019). We consider that exploring the ethical implications of both individual and group interactions on social media (such as WhatsApp) can shed new light not only on *how* young people choose to engage but also about *what* they decide to share about themselves and their lives.

Secondly, in this article, we have sought to query the status of the ‘absence’ of the researcher. In other words, how ‘absent’ is the researcher really despite their physical absence (perhaps compounded further by the bizarre situation in which the PI for our project was in the same city for some of the online research, but not able to be physically proximate to young people in Franco da Rocha)? Without entering into complex debates about the ‘there-ness’ of online spaces, or the ‘virtual/actual divide’ (Crang et al., 1999), it was the case that the PI was arguably *more* ‘present’ in virtual interactions than in face-to-face settings, despite being in a different country during part of the research. She was able to respond, either immediately or with a slight delay, to conversations on WhatsApp that could last for days or weeks. In fact, she sometimes felt compelled to respond at times when she was not ‘working’ (queueing the supermarket, for instance). This is not merely a practical methodological question but one that brings us full circle to some of the ethical questions that we posed earlier; specifically, might these forms of ‘presence’, which look little like traditional PAR methods, enable fruitful forms *of* participation and alleviate discomfort in dealing with sensitive issues affecting vulnerable groups? Certainly, a degree of invisibility, even anonymity, benefitted some participants. Meanwhile, other aspects became more visible or better able to be expressed – including in humorous and often fairly equal exchanges about food habits that were prompted by the PI’s being an outsider (a German researcher living and working in Brazil).

Thirdly, another lesson (and a challenge) was to ‘not always make it about us’ when participants did not respond. When we think about failures (Harrowell et al., 2018; Davies et al., 2021), it is easy to assume that a proposed activity was not sufficiently interesting or engaging. But what if participants were just busy or did not have access to a phone that week, were ill, or simply just forgot to respond? Moreover, arguably, this brings to bear a classic question for PAR scholars – that of *power* – in a rather different light: it felt to us that, at least in some situations, young people were in much more control when it came to their interactions with us. Non- or minimal response was not always a matter of lack of knowledge or lack of phone access; rather, by not responding, by responding later, or by choosing to respond through simple text messages, young people were able to dictate both when and how they responded, and to what. Whilst we might do our level best to ensure that vulnerable groups can withdraw from the research process when it takes place in-person, can we ever be sure that, *in the moment*, some participants do not feel pressured to respond, however, carefully we attempt to manage a group interaction? Not following the content of the group chats without actively engaging may also count as participation and a form of ‘silent expression’. In summary, the above scenarios denote a shift of power relations from the researcher to the participant who decides on whether, when and how to participate (while also considering the limitations of sometimes not being *able to* respond in a timely manner due to a lack of access to mobile devices). These shifting power relations potentially place the researcher in a position of vulnerability (Bashir, 2020).

Fourthly, we are not positing that digital methods are a solution to the question of equitable inclusion in research, nor to the (possible) problems of in-person PAR. However, they do, for us, provoke several challenges and opportunities – as outlined above – about what we mean when we talk about ‘participation’ in PAR. This is particularly the case in contexts where some vulnerable (young) people may prefer interactions that look little *like* PAR but enable them to participate in ways that *matter* to them (Horton and Kraftl, 2006).

Finally, to understand the future of PAR research, we need to engage with reflections that go beyond the role of digital methodologies for conducting qualitative research with vulnerable youth. Little has been written about how social media can become an opportunity for creating social relationships (Patulny and Seaman 2017; Décieux et al., 2019) and accessing a virtual reality beyond the socio-economic and spatial constraints of everyday conditions, especially for vulnerable young people in under-resourced and disadvantaged contexts. Therefore, it is not surprising that young people in the peripheral communities of fragmented and highly unequal urban metropolis such as Sao Paulo are quick to use social media channels such as WhatsApp as (alternative) ways of self-expression. In these virtual communication spaces, the young people set their own rules, thereby creating novel forms of legitimacy and (self-) expression. Yet, digital fatigue, the post-pandemic impacts of loneliness, and an increased need for human connection may drive a shift back to preference for face-to-face interactions. Based on these reflections, the future of PAR may as well be hybrid, pairing face-to-face interactions with the potentials of the digital to enable new ways of access, inclusion and meaningful expression that seek to empower groups in challenging conditions. Nonetheless, this article has opened out and critically evaluated several the key challenges, opportunities and considerations for digital research in terms of its complex, processual nature and the ways in which it may go beyond ‘compensation’ to facilitate diverse and contextually aware forms of ‘expression’.

## Notes

1. Postdoctoral Individual Global Fellowship funded by the European Union’s Horizon 2020 Research and Innovation Programme under the Marie Sklodowska-Curie Grant Agreement No. 833401 (08/2019-02/2023).

2. We use the terms ‘digital’ and ‘online’ research interchangeably.

3. Brazilian term for bush/forest/grass.

4. During our physical return to the field later in 2021, some of our participants preferred to continue interacting online instead of engaging in follow-up face-to-face activities. Hence, the pandemic may have exacerbated a growing tendency for substituting face-to-face contact with more mediated forms of interaction (Patulny and Seaman, 2017).
